# Transcranial Magnetic Stimulation for Major Depressive Disorder in Pregnancy: A Literature Review

**DOI:** 10.7759/cureus.5431

**Published:** 2019-08-19

**Authors:** Mansi R Shah, Alekhya Jampa, Mandeep Kaur, Chris A Robert, Rikinkumar S Patel

**Affiliations:** 1 Psychiatry, The Wright Center, Scranton, USA; 2 Obstetrics and Gynecology, KLE University, Jawaharlal Nehru Medical College, Belgaum, IND; 3 Psychiatry, American University of Antigua, Hayward, USA; 4 Obstetrics & Gynecology, California Institute of Behavioral Neurosciences and Psychology, Fairfield, USA; 5 Psychiatry, Griffin Memorial Hospital, Norman, USA

**Keywords:** tms, tms therapy, depression, major depressive disorder, safety, treatment efficacy, pregnancy

## Abstract

Major depressive disorder (MDD) is a growing problem among pregnant women as current treatment with antidepressants pose significant risks to the mother and fetus. Transcranial magnetic stimulation (TMS) is a neuromodulation technique that is being increasingly utilized to treat MDD in adults. We conducted a literature search using the keyword “TMS” and cross-referencing it with MDD, depression, major depressive episode, pregnancy, efficacy, safety, and clinical trial. This review explores current studies conducted to evaluate the efficacy and safety of TMS to treat MDD in pregnant females. Low-frequency TMS over the right dorsolateral prefrontal cortex, when given to pregnant women with MDD during the second and third trimester, has shown a significant response in depressive symptom reduction. TMS offers a promising alternative to current treatment options for managing MDD during pregnancy, but with limited research available, its safety and efficacy still need to be studied by conducting multicenter trials and long-term studies.

## Introduction and background

Major depressive disorder (MDD) during pregnancy is a major concern. It is estimated that 13% of women suffer from depression during pregnancy [1]. Pregnant females with MDD are less likely to seek prenatal care and are at higher risk of pregnancy-related complications (preterm birth, lower birth weight, and pre-eclampsia). The resulting maternal depression is known to have long-term impacts on maternal-infant bonding and child development and behavior [1-2].

There are various pharmacological treatment options available for MDD during pregnancy, but the risks associated with fetal exposure to psychotropic medications are high. Thus, many women choose not to take antidepressants during pregnancy [3]. Bernard N et al. study found a three-fold increased risk of pre-eclampsia in women taking antidepressants and/or anxiolytic medicine before 16 weeks of pregnancy [4]. There is a significantly increased risk for preterm birth (1.7 times) and low birth weight (1.4 times) with the use of antidepressants [5]. Use of antidepressants during pregnancy is associated with adverse fetal growth and birth outcomes including reduced fetal head growth by 0.18 mm per week and increased preterm birth risk by 1.2 times [6].

Transcranial magnetic stimulation (TMS) is conducted by delivering non-invasive magnetic pulses to the dorsolateral prefrontal cerebral cortex [3]. Magnetic pulses depolarize the neurons at deeper levels via trans-synaptic pathways and lead to the release of neurotransmitters. The pulses can be delivered at different frequencies to excite or inhibit cortical neurons [3]. TMS mostly activates inhibitory gamma-aminobutyric acid (GABA) neurons in the cortex, which results in depression of pyramidal cell glutamatergic output. Increased release of neurotransmitters increases blood flow and glucose metabolism in the regions that are activated, thus reducing depressive symptoms [3]. The high-frequency pulses to the right and low-frequency pulses to the right prefrontal cortex have shown to be effective in reducing depression in pregnant women [3].

The US Food and Drug Administration (FDA) approved TMS for adults with depression who have failed a single antidepressant trial in the current depressive episode. TMS has been an effective treatment for MDD in pregnancy for those who do not wish to be on psychotropic regimen [3, 7]. TMS for MDD in pregnant women has shown great potential, but its safety and efficacy still need to be further explored.

We conducted a literature search on Medline (January 1, 2010 to January 1, 2019) using the keyword “TMS” and cross-referencing it with MDD, depression, major depressive episode, pregnancy, efficacy, safety, and clinical trial. The goal of this article is to review current literature and explore the efficacy, safety, and contraindications for the use of TMS during pregnancy for MDD. 

## Review

Efficacy of TMS in pregnancy

The pilot study by Kim et al. was conducted in ten women with MDD in the second or third-trimester pregnancy [3]. The women were treated with 20 sessions of 1 Hz TMS at 100% motor threshold (MT) to the right dorsolateral prefrontal cortex (DLPFC), the area which is believed to be hypoactive in depression [3]. About 70% of women reported improvement of symptoms with more than 50% decrease in Hamilton depression rating scale (HDRS-17) scores. Approximately 30% of women met the criteria for remission treatment for MDD after the 20th TMS session [3].

This pilot study was followed by another randomized controlled trial by Kim et al. that looked at the effects of TMS on levels of progesterone and estradiol in pregnant women with MDD [8]. Subjects (N = 139) were between the 18-39 weeks and 14-34 weeks gestation with HDRS-17 score ≥18 and clinical global impression scale severity (CGI-S) ≥3. The results indicated a significant decrease in HDRS-17 scores compared to the control group, but no significant difference was noted for response and remission for treatment [8]. No significant differences were found between pre and post-intervention estradiol and progesterone. There were also no significant differences found in infant outcomes among the TMS and control groups [8].

Hizli Sayer et al. studied the effectiveness and safety of TMS use during pregnancy with 30 pregnant women receive repetitive TMS (rTMS) over the left prefrontal cortex for six days, and rTMS intensity at 100% MT [9]. TMS group had Hamilton depression rating scale (HAM-D) score decreased from 26.77 ± 5.58 to 13.03 ± 6.93 (P < 0.001) after 18 sessions of rTMS [9]. The treatment was well-tolerated, and no significant side effects were noted. Furthermore, 41.4% of the women reported improved mood symptoms, 20.7% of women had remission (HAM-D score below 8), 34.5% had partial remission, and only 3.4% had worsened HAM-D scores after TMS [9].

Safety of TMS in pregnancy

When administered with recommended guidelines, TMS is generally safe with few side effects and limited adverse effects [10]. The common side effects of TMS include localized pain on the site of stimulation, neck, and head pain. The pain is due to local stimulation of superficial nerves or facial muscle stimulation [10]. Those reported side effects are common, but not severe as only less than two percent of cases in clinical trials reported discontinuing the treatment due to pain. The localized pain at the site of treatment is seen to improve with treatment [10].

Adverse side effects are low in incidence and are significantly reduced when proper safety precautions are taken [10]. Some of the adverse effects include:

1. Seizures

The risk of seizures is low and thought to be similar to the incidence of seizures caused by antidepressant medications (0.1 - 0.6%). The risk of seizures increases with the higher frequency treatment protocol [10]. Other factors that may induce seizures include pre-existing neurological conditions, substance use, and changes in concurrent medications during TMS. Status epilepticus is not a side effect of TMS [10].

2. Hearing Impairment

There is a loud clicking sound produced during each TMS pulse. Thus, hearing protection is critical. Without proper hearing precautions, transient changes in auditory threshold have been noticed in humans, and permanent hearing damage has been seen in rabbits [10]. However, with proper hearing precaution, many studies have not found any significant hearing damage [10].

3. Cognition and neurodevelopment

Cognition impairment is a substantial concern with other neuromodulation techniques such as electroconvulsive therapy (ECT). However, current studies have not found any significant changes in perception with TMS [10].

4. Fetal outcomes

Eryilmaz et al. evaluated the impact of TMS on the neurodevelopment of fetus during the pregnancy using Ankara developmental screening inventory [11]. Study subjects were alive, born children of women who were treated with rTMS during pregnancy between the years of 2008 and 2013. Results showed that exposure to rTMS during pregnancy did not have a significant association with poor neurocognitive and poor motor development outcome [11]. Language delay was similar to that seen in offspring of rTMS treatment group and untreated group [11].

Contraindications of TMS in pregnancy

TMS should be restricted in patients with metal or electronic implants [10]. TMS is contraindicated in patients with cochlear electronic/magnetic implants that are in close contact with the TMS coil. Although the recommendation varies, the distance of the implant must be at least 10 cm from the coil [10].

## Conclusions

Currently, limited studies have been done to evaluate the short-term and long-term efficacy and safety of TMS during pregnancy for MDD. Past studies showed improvement in maternal functioning and depressive symptoms after being treated with TMS. Low-frequency TMS over the right DLPFC, when given to pregnant women with MDD during their second and third trimester, has shown a significant response in depressive symptom reduction. So, as TMS has been FDA approved for management for MDD, it can be considered as a safe option for pregnant women who are not willing to be on antidepressants. TMS offers a promising alternative to current treatment options for managing MDD during pregnancy, but with limited research available, its safety and efficacy still needs to be studied by conducting multicenter trials and long-term studies. 
